# A genetic study of immunity in depression and interactions with childhood maltreatment

**DOI:** 10.1038/s41398-026-03935-5

**Published:** 2026-03-19

**Authors:** Marisol Herrera-Rivero, Daniel L. McCartney, Heather C. Whalley, Klaus Berger, Andrew M. McIntosh, Bernhard T. Baune

**Affiliations:** 1 Department of Psychiatry, University of Münster; Joint Institute for Individualisation in a Changing Environment (JICE), University of Münster and Bielefeld University, Münster, Germany; 2 Centre for Genomic and Experimental Medicine, Institute of Genetics and Cancer, University of Edinburgh, Edinburgh, UK; 3 Centre for Clinical Brain Sciences, University of Edinburgh, Edinburgh, UK; 4 Institute of Epidemiology and Social Medicine, University of Münster, Münster, Germany; 5 Department of Psychiatry, Melbourne Medical School, The University of Melbourne; The Florey Institute of Neuroscience and Mental Health, The University of Melbourne, Melbourne, Australia

**Keywords:** Genomics, Depression

## Abstract

Genetic and environmental factors contribute to depression. Among the latter, early life adversity and immune dysregulation have been consistently linked with depression. Childhood maltreatment (CM) is believed to induce immune dysregulation later in life. However, it is not known how CM might interact with genetic immune factors to contribute to the occurrence of depression. We investigated how genetic variability in 2370 genes from 20 immune pathways associates with a broadly-defined lifetime depression phenotype at gene- and pathway-level, and how this variability interacts with CM. Depression analysis was carried out in 13,309 individuals (1867 cases) from Generation Scotland (GS). CM interaction analysis was carried out in a subset of 749 individuals (99 cases) from GS and an independent sample of 509 individuals (96 cases) from the German BiDirect (BD) Study for which both genetic and CM data was available. Interactions with different types of CM were tested using the subscales of the childhood trauma questionnaire (CTQ). These results were meta-analyzed to obtain general gene-CM interactions. We found association of the *GHR* gene (false discovery rate –FDR– = 0.03, z = 4.2) and Reactome “RUNX1-regulated transcription of genes involved in myeloid cell differentiation pathway” (FDR = 0.016, beta = 1.2) with depression in GS. After meta-analysis, 56 immune gene-CM interactions were associated with depression (FDR < 0.05) in both GS and BD. These exert functions in hematopoiesis, pathogen recognition and stress responses, among others. Network analysis suggested macrophages as main expressing cell types. Our results underscore the involvement of hematopoietic alterations and immune gene-CM interactions in the development of depression.

## Introduction

Many genetic and environmental factors modify the risk for depression. In particular, exposure to early life adversity and immune dysregulation have been independently and consistently associated with depression [1, 2]. Beyond observations of altered molecular and cellular markers of immune activity in depressed individuals, a causal role for immune dysfunction in depression is suggested by genetic findings from genome-wide association (GWA) and mendelian randomization studies [3]. Nevertheless, there is an incomplete understanding of the molecular mechanisms involved. On the other hand, childhood maltreatment (CM), which is the exposure to early life trauma, such as physical and emotional abuse or neglect, is the most significant environmental factor associated with the risk for depression in adulthood [4]. CM leads to immune dysregulation later in life. This has been evidenced, for example, by altered levels of inflammatory cytokines and of transcripts of molecular markers of immune function [5, 6]. In addition, specific types of trauma have shown the capacity to differentially affect immune profiles [7]. Therefore, it is proposed that CM increases vulnerability to depression due to its effects on immune activity [8]. However, because not all individuals exposed to CM develop depression, it is reasonable to assume that other factors contribute to modulate the outcome.

We hypothesized that interactions between immune genetic factors and CM favor the development of depression. To test this hypothesis, we studied 20 representative immune pathways (including 2370 protein-coding genes) in participants of the Generation Scotland cohort and the German BiDirect Study, investigating gene- and pathway-level genetic variability associated with lifetime depression and its interactions with CM. Our approach aimed at i) reducing the burden of multiple testing by selecting specific immune pathways that we considered representative of a broad spectrum of immune processes commonly involved in disease, ii) capturing a larger spectrum of (unipolar) depression syndromes by employing a broad definition of the phenotype, and iii) improving reproducibility by focusing on the combined effects of variants within genes and pathways instead of single genetic variants.

## Methods

### Study population

Generation Scotland (GS) is a large, longitudinal genetic epidemiology study of the Scottish population commenced in 2006. It comprises over 30,000 volunteers and includes a large family-based component, the Scottish Family Health Study (GS:SFHS), with over 24,000 individuals from 7000 families. Details can be found on the study’s website (www.generationscotland.org) and previous publications [9]. Here, we used a GS sample of 13,309 non-first-degree relatives, including 1867 individuals with lifetime depression, to study genetic associations with depression. Because GS:SFHS is a family study, excluding first-degree relatives from our study sample ensured a better representation of the general Scottish population. CM data for interaction analyses was available for 749 of these individuals, including 99 individuals with lifetime depression. Depression cases were defined using a broad phenotype definition based on ICD-10 (International Classification of Diseases 10th Revision) codes and self-report as individuals with a) a depressive episode (ICD-10: F32, n = 235), and/or b) a diagnosis of recurrent major depressive disorder (ICD-10: F33, n = 17), and/or c) self-reported depression (n = 1749), and d) no diagnosis of bipolar disorder (ICD-10: F31). Controls were defined as individuals with none of the above.

BiDirect is a longitudinal study initiated in 2009, designed with the aim to explore the relationship between depression and subclinical arteriosclerosis. It comprises over 2300 individuals distributed in three cohorts: 1) depression patients hospitalized at the time of recruitment, 2) patients after an acute coronary event, and 3) randomly sampled community-dwelling adults. All participants were recruited in the district of Münster, Germany, and underwent extensive, repeated phenotyping during a period of 10 years. The study design and methods have been described elsewhere [10]. For CM interaction analyses, we used a BD sample of 509 participants from the general population (i.e. cohort 3) with CM data, including 96 individuals with a lifetime history of depression. These represented a better match to the study design found in GS, given that the depression cohort in BD is enriched for current severe forms of the disorder due to recruitment during an episode with need of hospitalization.

All participants provided written informed consent. Methods were carried out in accordance with the ethical standards laid down in the Declaration of Helsinki. GS:SFHS received ethical approval from the NHS Tayside Committee on Medical Research Ethics (REC Reference Number: 05/S1401/89). The BiDirect Study was approved by the ethics committee of the University of Münster and the Westphalian Chamber of Physicians in Münster, North-Rhine-Westphalia, Germany.

### Selection of genes/pathways

Here, applying a hypothesis-driven approach, 20 pathway sets of innate and adaptive immunity processes were considered by the authors representative of a broad spectrum of immune processes commonly associated with disease and were ultimately selected as pathways of interest for the study. The gene lists of the following Reactome pathways were downloaded from the curated gene sets (C2) human collection at MSigDB (Supplementary Table 1): Adaptive immune system (M1058), Cellular response to chemical stress (M41836), Cellular responses to stimuli (MM15548), Chemokine receptors bind chemokines (M625), Class I MHC-mediated antigen processing & presentation (M1066), Complement cascade (M19752), Cytokine Signaling in Immune system (M1060), Diseases of Immune System (M27428), Immunoregulatory interactions between a Lymphoid and a non-Lymphoid cell (M8240), Inflammasomes (M1072), Innate Immune System (M1036), Interferon Signaling (M983), MHC class II antigen presentation (M705), Neutrophil degranulation (M27620), Regulation of TLR by endogenous ligand (M27571), RUNX1 regulates transcription of genes involved in differentiation of myeloid cells (M27799), Signaling by Interleukins (M874), Signaling by the B Cell Receptor (BCR) (M608), TCR signaling (M15381), and Toll-like Receptor Cascades (M7494). The genomic coordinates for the start and end positions of a total of 2417 pathway genes were annotated using the Ensembl’s hg19 genome build.

### Genotype data

This study analyzed genotype datasets from GS and BD. Sample collection and processing as well as genotyping and genotype quality control (QC) procedures in GS [11, 12] and BD [10, 13, 14] have been described elsewhere. Both genotype datasets were imputed using the Haplotype Reference Consortium (HRC) panel. Post-imputation QC steps were applied using plink 1.9 or 2 [15] and the R package plinkQC. These included the removal of single nucleotide polymorphisms (SNPs) with Rsq<0.8, MAF < 1% and Hardy-Weinberg equilibrium p < 1 × 10^−6^. Individuals who failed the heterozygosity test were removed in both datasets. In GS, we excluded one of a pair of individuals with PI-HAT > 0.5 (first-degree relatives), with preferential exclusion of controls over cases. The BD dataset already consisted only of unrelated individuals. After QC, we extracted from these datasets the selected immune pathway genes using the start and end positions of each gene with a window of ± 1 kb in the hg19 genome build. The analyzed datasets contained 312,653 variants corresponding to 2376 genes in GS, and 288,264 variants corresponding to 2371 genes in BD.

### Immune gene and pathway associations with depression

Gene- and pathway-level association testing in GS was conducted using the Multi-marker Analysis of GenoMic Annotation (MAGMA) software [16]. Here, we applied the multi-model for gene-level analysis, which computes a principal components regression model, a SNP-wise mean model and a SNP-wise top 1 model to test for association of genes with the phenotype, and aggregates the resulting gene p-values. Subsequently, the pathway-level analysis utilizes the aggregated gene p-values to test for association between the defined pathway sets and the phenotype. We adjusted the gene model for age, sex, and the first five dimensions coming from a principal components analysis (PCA) of the genotypes. Statistical significance for both analyses was set to false discovery rate (FDR) < 0.05.

### Interactions with childhood maltreatment

The Childhood Trauma Questionnaire (CTQ) was used for screening of history of CM among GS and BD participants. Here, we used the rank-normalized total scores obtained for each of the five CTQ subscales: emotional abuse (EA), physical abuse (PA), sexual abuse (SA), emotional neglect (EN) and physical neglect (PN). Gene- and pathway-level association analyses were conducted using MAGMA as described above, with the addition of an interaction component that tested for interactions between the genes and the CTQ variables while computing gene-level associations with the phenotype. Therefore, five gene- and five pathway-based association tests, one for each CTQ subscale, were performed in both datasets. Following an interaction analysis, the pathway-level analysis in MAGMA utilizes the p-value coming from the full gene model (i.e. main gene effects + interaction effects). In addition, to obtain an integrative view of CM interactions that favor the development of depression, we combined the (full model) p-values obtained for the independent CTQ subscales by applying the Fisher’s method using the metap R package. Statistical significance was set to FDR < 0.05.

### Network and enrichment analysis

To better inform the biological context of our findings from the CM interaction analysis, we used STRING v12.0 [17] to create a protein-protein interaction (PPI) network from those genes that showed significant gene-CM interactions in GS and BD. This network was set to include only high confidence interactions (score ≥0.7). We hid unconnected nodes and extended the network by 10 non-query interactors in the second shell (white nodes) to fully connect the remaining interactions. Finally, we analyzed this network for overrepresentation of Gene Ontology biological processes (GO_BPs) and expression tissues over a background of the whole genome. Overrepresented terms were considered those with FDR < 0.05 and strength>1.

## Results

**Fig. 1:**
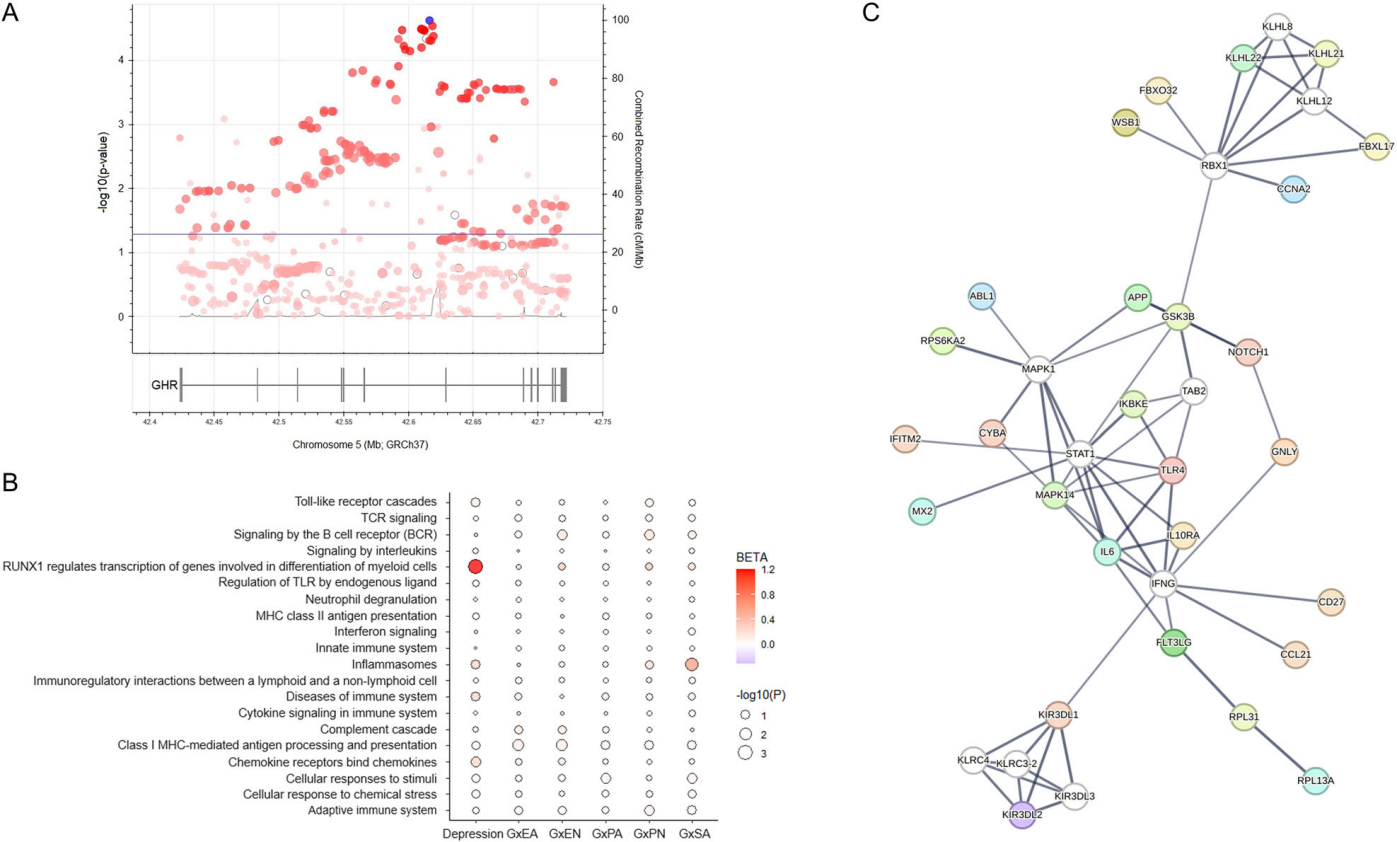
Overview of main results. **A** Regional association plot of the *GHR* gene in GS. The blue dot represents the top SNP in the region (rs10440649, p = 2.37 × 10^−5^, odds ratio=1.21). The blue line depicts the threshold for nominal significance (p = 0.05). The intensity of the red-colored dots illustrates linkage disequilibrium (LD) with the top SNP, where more intense colored dots are in higher LD with the top variant. **B** Pathway-level analysis results in GS. **C** PPI network obtained with STRING for the 56 replicated immune gene-CM interaction associations with depression. White nodes indicate non-query proteins. Edge thickness outlines the strength of the PPIs. G gene, EA emotional abuse, PA physical abuse, SA sexual abuse, EN emotional neglect, PN physical neglect.

First, we studied gene- and pathway-level associations with lifetime depression of 2376 genes annotated to 20 curated immune pathways (Supplementary Table 1) in 13,309 individuals from GS. This sample contained 1867 (14%) depression cases with a mean age of 47.5 ( ± 12.8) years and high proportion of females (70.8%), and a control group of 11,442 individuals of similar age (Table 1). Using the multi-model analysis in MAGMA, we identified one immune pathway gene, the growth hormone receptor (*GHR*; FDR = 0.03, Z = 4.2), and one immune pathway, the Reactome “RUNX1 regulates transcription of genes involved in differentiation of myeloid cells” (FDR = 0.016, beta = 1.2), associated with depression. Three (*CSF2*, *RUNX1* and *PRKCB*) of the seven genes annotated to this pathway were tagged as potentially responsible (p < 0.05) for the observed association. The SNP-level association results for *GHR* are shown in Fig. 1A. We also show in Table 2 the top results (FDR < 0.25) from the gene association analysis that were not considered statistically significant. These included 10 genes annotated to the cytokine signaling, cellular responses to stimuli, interferon signaling and adaptive immune system pathways, among others. Results from the pathway-level association analysis with depression are shown in Fig. 1B.

**Table 1:** Basic characteristics of the population-based Generation Scotland (GS) and BiDirect samples used in the study.

	All	Depression	Control
*GS sample*			
N	13,309	1867	11,442
Females (%)	7874 (59.2)	1322 (70.8)	6552 (57.3)
Age (mean ± SD)	47.3 ± 14.9	47.5 ± 12.8	47.3 ± 15.2
*GS - CTQ subsample*			
N	749	99	650
Females (%)	437 (58.3)	69 (69.7)	368 (56.6)
Age (mean ± SD)	52.3 ± 10.2	50.1 ± 10.3	52.6 ± 10.1
*BiDirect (CTQ sample)*			
N	509	96	413
Females (%)	253 (49.7)	64 (66.7)	189 (45.8)
Age (mean ± SD)	54.1 ± 8	54.2 ± 7.4	54.1 ± 8.2

**Table 2:** Top findings (FDR < 0.25) from the gene-level association analysis with depression in Generation Scotland.

Gene	# SNPs	# Parameters	Z	p-value	FDR	Pathway(s)
*GHR*	575	52	4.21	1.28E-05	0.0304	Cytokine signaling
*BTN3A2*	112	12	3.84	6.19E-05	0.0735	Adaptive immune system. Inhibits the release of IFNG from activated T-cells
*HIRA*	216	20	3.45	2.79E-04	0.2213	Cellular responses to stimuli
*TUBB4A*	21	11	3.26	5.57E-04	0.2478	Adaptive immune system; cellular responses to stimuli; cytokine signaling; interferon signaling; MHC class II antigen presentation
*PHC3*	129	24	3.24	5.93E-04	0.2478	Cellular responses to stimuli
*LILRA4*	31	12	3.20	6.98E-04	0.2478	Immunoregulatory interactions between lymphoid and non-lymphoid cells; adaptive immune system
*ADAR*	95	13	3.17	7.65E-04	0.2478	Cytokine siganling; interferon signaling
*SKP2*	104	17	3.11	9.34E-04	0.2478	Adaptive immune system; cellular responses to stimuli; cellular responses to chemical stress; class I MHC mediated antigen processing presentation
*FNIP1*	207	19	3.11	9.39E-04	0.2478	Cellular responses to stimuli
*CSF2*	9	5	3.08	1.05E-03	0.2488	Cytokine signaling; interleukin signaling; RUNX1 regulates transcription of genes involved in differentiation of myeloid cells
*COL17A1*	91	23	3.05	1.15E-03	0.2488	Immunoregulatory interactions between lymphoid and non-lymphoid cells; adaptive immune system

**Fig. 2:**
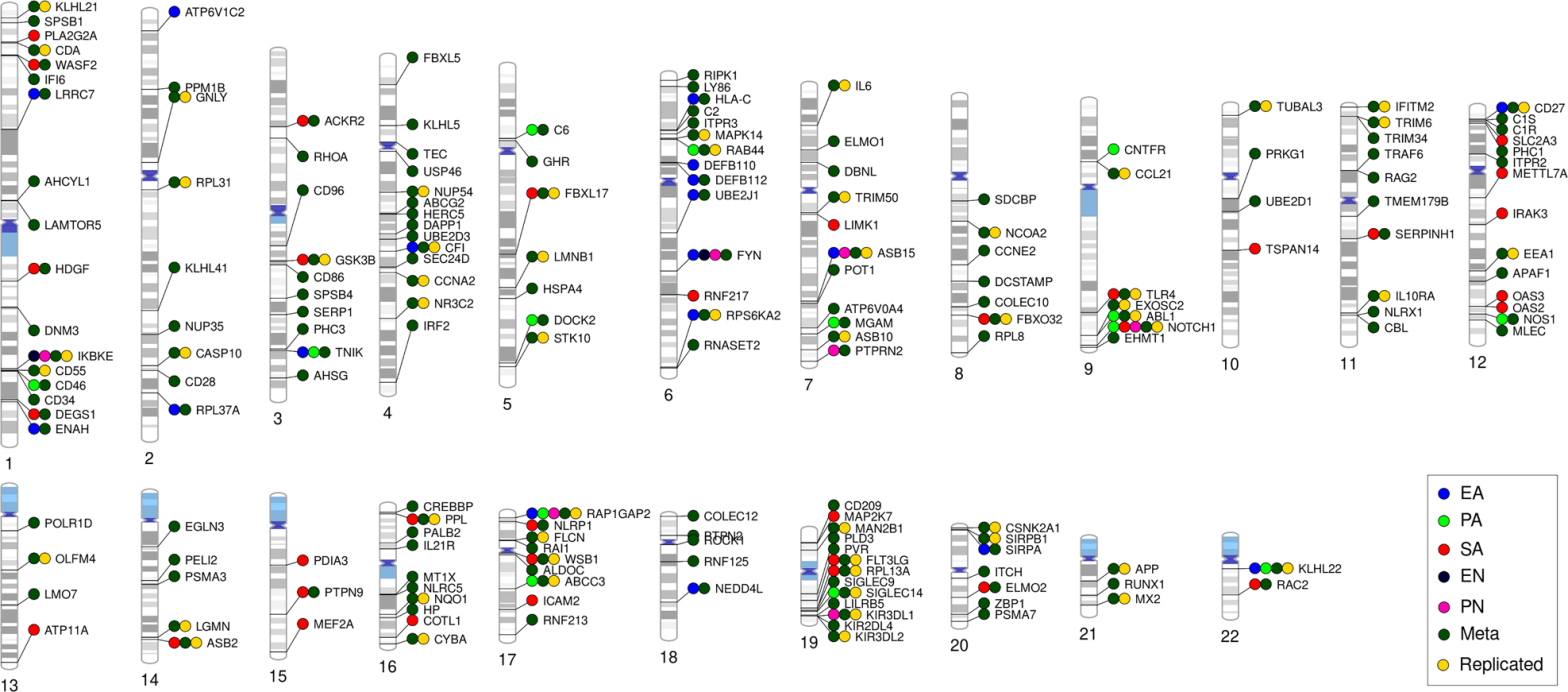
Phenogram summarizing the most robust findings from the immune gene-CM interaction association analysis with depression. Overlaps between significant findings in GS (FDR < 0.05) and nominal observations in BD (p < 0.05) are shown, as well as the replicated (FDR < 0.05 in both independent datasets) findings. EA emotional abuse, PA physical abuse, SA sexual abuse, EN emotional neglect, PN physical neglect, meta: meta-analysis of CTQ subscales.

Second, we investigated the association of gene-CM interactions with depression using the CTQ in a subsample of 749 GS participants (5.6% from study sample; mean age: 52.3 ± 10.2 years, 58.3% females), including 99 (13.2% from CM subsample) depression cases, and an independent sample of 509 individuals from the population-based cohort of BD (mean age: 54.1 ± 8 years, 49.7% females), including 96 (18.9% from CM subsample) depression cases (Table 1). All individuals in the CM subsamples had data for all CTQ subscales. The analyses using the different CTQ susbcales in GS identified 122 immune pathway genes that interacted with the total EA scores in association with depression (Supplementary Table 2), 127 that interacted with total PA (Supplementary Table 3), 416 with total SA (Supplementary Table 4), 10 with total EN (Supplementary Table 5) and 42 with total PN (Supplementary Table 6). However, these analyses in BD resulted in almost only nominal associations that did not survive correction for multiple comparisons. Therefore, we decided to look-up the significant findings from GS in BD using a threshold of p < 0.05. With this, we found 18 (14.8%) overlaps for EA, 14 (11%) for PA, 34 (8.2%) for SA, 2 (20%) for EN and 7 (16.7%) for PN between both samples (Table 3, Fig. 2, Supplementary Table 2-6). The most significant GS genes interacting with each CTQ subscale that showed nominal significance in BD were: *ASB15* in EA (FDR_GS_ = 0.0026, p_BD_ = 0.0131), *TNIK* in PA (FDR_GS_ = 7.7x10^−4^, p_BD_ = 0.002), *SERPINH1* in SA (FDR_GS_ = 2.1x10^−4^, p_BD_ = 0.03), *FYN* in EN (FDR_GS_ = 2.3x10^−4^, p_BD_ = 0.0423) and *IKBKE* in PN (FDR_GS_ = 0.0033, p_BD_ = 0.0041).

**Table 3:** Summary of findings from gene-CM interaction associations with depression in GS and BD.

	GS	BD		GS FDR < 0.05	GS & BD
	FDR < 0.05	p < 0.05	FDR < 0.05	& BD p < 0.05	FDR < 0.05
Total EA	122	285	2	18	0
Total PA	127	240	0	14	0
Total SA	416	236	0	34	0
Total EN	10	238	2	2	0
Total PN	42	254	0	7	0
CTQ (meta)	653	563	173	167	56

After, we combined the p-values obtained for the CTQ subscales into one p-value that should reflect a general association of gene-CM interactions with depression, regardless of the type of trauma. We found 653 significant (FDR < 0.05) genes in GS (Supplementary Table 7). Because we had adopted the look-up approach using a p < 0.05 criterium for BD before, we applied the same strategy here and found 167 (25.6%) overlaps with GS (Supplementary Table 7). Importantly, we found that 56 from these genes were statistically significant (FDR < 0.05) also in BD (Tables 3–4, Fig. 2) and, therefore, these gene-CM interactions in association with depression were considered replicated in our study.

**Table 4:** CTQ-interacting immune pathway genes identified by meta-analysis in Generation Scotland that were replicated in BiDirect.

Gene	Generation Scotland		BiDirect		Functional roles (GeneCards)
	p-combined	FDR	p-combined	FDR	
*ASB15*	9.75E-20	5.80E-16	7.96E-05	9.93E-03	Ubiquitination and proteasomal degradation
*IKBKE*	8.31E-16	1.41E-12	3.20E-04	2.71E-02	Inflammatory response
*ABL1*	7.13E-14	7.71E-11	2.56E-04	2.30E-02	Cell growth and differentiation; apoptosis; response to stress; others
*RAP1GAP2*	1.80E-13	1.53E-10	3.44E-06	9.65E-04	Platelet function
*NOTCH1*	1.44E-11	6.44E-09	2.20E-07	1.37E-04	Cell fate determination
*CD27*	2.99E-08	4.51E-06	1.63E-04	1.67E-02	Generation and long-term maintenance of T cell immunity
*GNLY*	7.57E-08	9.31E-06	5.00E-04	3.75E-02	Antimicrobial activity against intracellular pathogens
*RPS6KA2*	1.58E-07	1.75E-05	5.35E-05	7.55E-03	Cell growth and differentiation
*EEA1*	2.61E-07	2.69E-05	6.54E-04	4.64E-02	Endosomal trafficking
*GSK3B*	2.88E-07	2.93E-05	5.29E-07	2.72E-04	Glucose homeostasis; inflammation; ER-stress; apoptosis; others
*FBXO32*	3.44E-07	3.38E-05	1.40E-06	5.53E-04	Ubiquitination and proteasomal degradation
*KLHL22*	3.54E-07	3.42E-05	1.49E-06	5.55E-04	Cell growth; protein metabolism; cytokinesis
*FLT3LG*	3.81E-07	3.60E-05	8.71E-09	1.03E-05	Proliferation of early hematopoietic cells
*EXOSC2*	6.19E-07	5.37E-05	1.30E-04	1.45E-02	RNA processing and degradation events; cell growth
*WSB1*	8.13E-07	6.48E-05	1.66E-06	5.55E-04	Ubiquitination and proteasomal degradation
*PPL*	1.08E-06	8.36E-05	1.06E-06	4.65E-04	Possible localization signal in AKT1-mediated signaling
*CASP10*	1.99E-06	1.38E-04	6.87E-04	4.75E-02	Apoptosis
*KIR3DL1*	2.65E-06	1.77E-04	1.75E-05	3.34E-03	Inhibition of natural killer cell activity
*TRIM6*	3.53E-06	2.15E-04	6.88E-04	4.75E-02	Activation of IKBKE-dependent type I interferon signaling
*RAB44*	5.80E-06	3.27E-04	1.93E-05	3.58E-03	Calcium ion binding
*RPL13A*	6.41E-06	3.51E-04	8.19E-05	1.01E-02	Repression of inflammatory genes
*FLCN*	9.44E-06	4.88E-04	2.30E-04	2.14E-02	Response to nutrients; stem cell differentiation; others
*TRIM50*	1.03E-05	5.26E-04	4.53E-06	1.14E-03	Starvation-induced autophagy and NLRP3 inflammasome activation
*KIR3DL2*	1.63E-05	7.71E-04	2.56E-05	4.33E-03	Negative regulation of NK and T cell effector functions
*CD55*	1.78E-05	8.32E-04	1.91E-05	3.58E-03	Complement cascade
*RPL31*	1.86E-05	8.63E-04	3.98E-05	6.03E-03	Protein synthesis
*FBXL17*	1.92E-05	8.82E-04	2.82E-05	4.65E-03	Ubiquitination and proteasomal degradation
*CYBA*	3.07E-05	1.32E-03	2.77E-04	2.45E-02	Microbicidal activity of phagocytes
*SIGLEC14*	4.06E-05	1.65E-03	1.43E-04	1.54E-02	Cell adhesion
*ASB10*	4.56E-05	1.83E-03	3.93E-04	3.17E-02	Ubiquitination and proteasomal degradation
*CFI*	6.39E-05	2.39E-03	3.58E-07	2.02E-04	Complement cascade
*TLR4*	6.52E-05	2.43E-03	5.88E-07	2.90E-04	Pathogen recognition and activation of innate immunity
*TUBAL3*	7.48E-05	2.70E-03	3.47E-05	5.36E-03	Microtubule cytoskeleton organization and mitotic cell cycle
*NCOA2*	9.19E-05	3.21E-03	5.55E-08	4.38E-05	Coactivation of nuclear hormone receptors
*IFITM2*	9.54E-05	3.31E-03	2.69E-04	2.39E-02	Interferon-induced antiviral activity
*NR3C2*	1.38E-04	4.30E-03	5.25E-04	3.89E-02	Response to mineralocorticoids and glucocorticoids
*ASB2*	1.51E-04	4.65E-03	3.69E-04	3.04E-02	Ubiquitination and proteasomal degradation; hematopoiesis; others
*CSNK2A1*	1.63E-04	4.99E-03	6.02E-04	4.29E-02	Cell cycle control; transcription; circadian rhythm; apoptosis, others
*MX2*	2.34E-04	6.73E-03	2.03E-04	1.98E-02	Interferon-induced antiviral activity
*CCNA2*	3.07E-04	8.35E-03	4.93E-05	7.22E-03	Cell cycle control
*MAPK14*	3.41E-04	9.13E-03	3.58E-06	9.65E-04	Cellular responses to stimuli (e.g. pro-inflammatory cytokines, physical stress)
*OLFM4*	3.83E-04	1.01E-02	1.34E-04	1.47E-02	Cell growth and proliferation
*KLHL21*	4.69E-04	1.19E-02	6.51E-05	8.76E-03	Ubiquitination; cytokinesis
*NQO1*	4.98E-04	1.25E-02	1.69E-06	5.55E-04	Cellular redox state regulation
*LGMN*	5.16E-04	1.29E-02	3.50E-06	9.65E-04	Cell proliferation; antigen presentation; others
*IL10RA*	7.23E-04	1.68E-02	1.30E-04	1.45E-02	Inhibition of inflammation and starvation-induced autophagy
*LMNB1*	8.66E-04	1.96E-02	4.96E-04	3.75E-02	Nuclear activities
*APP*	1.28E-03	2.66E-02	4.24E-04	3.35E-02	Neurite growth, neuronal adhesion and axonogenesis; synaptogenesis; axonal transport; transcription regulation; copper homeostasis/oxidative stress; others
*CCL21*	1.39E-03	2.86E-02	5.44E-04	3.98E-02	Hematopoiesis; chemotaxis
*STK10*	1.61E-03	3.25E-02	2.11E-05	3.85E-03	Lymphocyte migration; cell cycle regulation
*IL6*	2.19E-03	4.13E-02	1.54E-04	1.60E-02	Inflammation; B cell maturation; T cell differentiation; metabolism; others
*ABCC3*	2.36E-03	4.39E-02	3.24E-06	9.61E-04	Protein transport
*SIRPB1*	2.45E-03	4.52E-02	2.00E-04	1.98E-02	Myeloid cell activation; others
*CDA*	2.52E-03	4.63E-02	2.21E-05	3.91E-03	Pyrimidine salvaging
*MAN2B1*	2.52E-03	4.63E-02	2.17E-04	2.08E-02	Cellular metabolism
*NUP54*	2.74E-03	5.00E-02	7.15E-05	9.42E-03	Trafficking across the nuclear membrane

In addition, we used PPI network analysis to better inform the biological context of the 56 replicated gene-CM interactions favoring depression (i.e. query proteins). The resulting network (Fig. 1C) had a PPI enrichment p = 2.18x10^−7^, 138 PPI pairs, and 37 connected proteins from a total of 66 nodes. The hub nodes in the network were all non-query proteins: IFNG and STAT1 (with 20 interactions each), followed by RBX1 (18 interactions) and MAPK1 (16 interactions). Enrichment analysis identified 78 GO_BPs and 31 tissues of expression that were overrepresented (FDR < 0.05, strength>1) in our network (Supplementary Table 8). Here, we were interested in observing enrichments of biological processes that were different from those pathways initially selected for our study, as well as of specific tissues and cell types expressing the identified genes. As expected, most of the GO_BP terms overrepresented in our network analysis were related to the original Reactome pathways included in the study, such as cellular responses to biotic stimulus, response to interferons, regulation of innate immune response and chemokine production, etc. However, we found some GO_BPs that provided more defined insights and some hints of effects beyond peripheral immunity including, for example, Astrocyte differentiation (FDR = 6.11x10^−5^), Cellular response to amyloid-beta (FDR = 0.0026), Regulation of long-term synaptic potentiation (FDR = 0.0035), Response to L-glutamine (FDR = 0.014), and various terms related to oxidative stress (e.g. Response to reactive oxygen species, Positive regulation of nitric-oxide synthase biosynthetic process, Positive regulation of oxidative stress-induced cell death, Positive regulation of reactive oxygen species metabolic process, etc.), the production of particular interleukins (IL-6, 1β, 17, −12 and −23), and stem cell differentiation, including Regulation of hematopoietic progenitor cell differentiation (FDR = 0.028) and Regulation of pro-B cell differentiation (FDR = 0.0305). In general, the network proteins showed enriched expression in peripheral macrophages and microglial cells (Supplementary Table 8). Nevertheless, we also found overrepresentation of other types of white blood cells, including B-lymphocytes, natural killer cells, helper and memory T-lymphocytes, monocytes and neutrophils. Interestingly, there was also enrichment for expression in the blood-brain barrier (BBB; FDR = 0.0124).

Finally, we performed pathway-level association analyses using the CTQ interaction gene results. However, we found only nominal associations in GS that did not survive correction for multiple comparisons (Fig. 1B). These were with the “Inflammasomes” (p = 0.0044, beta=0.6) and “Cellular responses to stimuli” (p = 0.04, beta=0.084) pathways in the SA analysis, the “Class I MHC-mediated antigen processing & presentation” pathway in the EN (p = 0.019, beta = 0.13) and EA (p = 0.024, beta=0.12) analyses, the “Adaptive immune system” (p = 0.038, beta = 0.084) and Signaling by BCR (p = 0.042, beta = 0.18) pathways in the PN analysis, and the “Cellular responses to stimuli” (p = 0.048, beta = 0.078) pathway in the PA analysis. After meta-analysis of CTQ subscales, the top pathway for this CM analysis in GS was the “Class I MHC-mediated antigen processing & presentation” (p = 5.8x10^−4^, FDR = 0.058). This was followed by the “Adaptive immune system” (p = 0.0168), Signaling by BCR (p = 0.0215) and “Cellular responses to stimuli” (p = 0.0476) pathways. In BD, there were no significant findings from the pathway-level analyses.

## Discussion

We conducted a candidate study of immune genetic factors in depression. Twenty immune pathways were studied. Associations with lifetime depression were tested at the gene- and pathway-level in a large cohort. In addition, interactions with different types of CM and with CM in general were investigated in two independent samples. We found association of the *GHR* gene and the Reactome “*RUNX1*-regulated transcription of genes involved in myeloid cell differentiation” pathway with depression, as well as a set of 56 genes that most robustly interacted with CM in depression and have implications for hematopoiesis and stress responses, among other immune processes. Network analysis of these 56 genes revealed further biological roles in brain function and important expressing cells, particularly macrophages. Our study underscored the role of hematopoietic alterations in depression, and supported the hypothesis that interactions between immune genetic profiles and CM contribute to disease development.

A repeatedly reported link between depression and immune dysfunction is underpinned by genetic studies that suggest a causal role of immune dysregulation in the development of depression [3, 18, 19]. Although the underlying molecular mechanisms governing this association are complex and yet not fully elucidated, it is believed that understanding the immune mechanisms that contribute to depression might aid in the development of novel therapeutic approaches [19]. It is well-documented that the brain possesses specialized immune-active cells, including microglia, which show macrophage-like functions, the perivascular, meningeal and choroid plexus macrophages, and lymphoid cells. Peripheral macrophages and T cells are also able to infiltrate the brain under certain conditions. In addition, cytokines are produced in the brain to regulate a multitude of functions, including mood and cognitive function, and peripheral cytokines reaching the brain have been shown to exert effects on behavior [1]. The presence of neuroinflammation as well as abnormalities in the levels of peripheral cytokines and numbers of immune cells in at least subsets of depression patients have been largely documented [1, 18, 20–22]. In our study, the associations of the *GHR* gene and the Reactome “*RUNX1*-regulated transcription of genes involved in myeloid cell differentiation” pathway with depression in GS provide evidence for a role of macrophage activation and myeloid cell production in the development of depression. GHR is well-known for its role in somatic growth in response to growth hormone (GH). However, it is also a cytokine receptor expressed by many cells, including macrophages, which makes macrophages responsive to GH. It has been suggested that GH is able to prime and activate macrophages in a manner similar to that observed with interferon-gamma (IFNγ) [23]. Moreover, the binding of GH to GHR on hematopoietic cells has been shown to regulate their proliferation and differentiation [24]. Few studies have suggested an involvement of GH in depression [25, 26].

It is believed that prolonged physical and psychological stress leads to long-lasting immune changes in the brain and periphery that contribute to depression. Indeed, the immune system plays a pivotal role in stress responses. Stress induces the activation of microglia, the NLRP3 inflammasome pathway, and the hypothalamic-pituitary-adrenal (HPA) axis that regulates glucocorticoids. It also induces immunosuppression and changes in the BBB, leading to brain infiltration of T cells and monocytes, among other effects [27]. The presence of stressors during sensitive periods of brain development, such as different types of childhood trauma, has been linked to immune dysfunction later in life, together with an increased risk of mental health problems, including depression [28–31]. Previous genetic studies have revealed interaction effects between genes related to neurological systems of stress and emotional responses and CM on depression and depression-related intermediate phenotypes, such as HPA activity and regional brain volumes [32], as well as between some immune genes (e.g. *IL6*, *CRP*, *TNF*) and CM on depression and/or depressive symptoms [33]. However, SNP-level findings have been largely inconsistent between studies. Using a gene-level approach to study interaction effects between immunity and CM on depression, we intended to overcome this challenge, at least to some extent. Although we observed substantial differences between the results obtained in GS and BD for the different types of CM, with only an overall overlap of 10.5% in these using a nominal threshold for BD, we found 56 genes (2.4% from all included in the study) that showed statistical significance in both datasets after meta-analysis. This replication in an independent sample evidenced credible immune gene-CM interactions associated with depression that suggested a role of macrophages and the regulation of processes related to the production of immune cells, the responses to oxidative stress and interferons, and sypnaptic functions as potential mechanisms underlying the relationship between immunity, CM and depression, all of which are in agreement with the literature. Despite the encouraging data, the results from our study remain to be validated in larger samples.

In addition, genes associated with Alzheimer’s disease (AD) and the cellular responses to amyloid-β (Aβ, a major role player in the pathobiology of AD), including *APP*, *NOTCH1*, *KIR3DL2*, *GSK3β* and *TLR4*, were found within the replicated set of genes in our CM interaction analysis. AD is strongly associated with immune dysfunction [34, 35]. Comorbidity of AD and depression is frequent, where depression may represent either a risk factor for AD or a prodromal stage of the disease [36, 37]. Various studies have identified common pathways between depression and AD, including dysregulation of the HPA axis, inflammation and Aβ accumulation, a hallmark of AD pathophysiology, in patients with depression [36, 38–40]. Moreover, CM has also been suggested as a risk factor for AD [41]. Therefore, our study adds evidence in support of the relationship between CM, immunity, depression and AD, and pinpoints specific candidate genes.

Of note, because we used the population-based cohort of BD due to its more similar characteristics in comparison to GS, we performed a secondary analysis to explore whether the identified associations were also significant when comparing the depression cohort of BD (i.e. cohort 1) against a subgroup of individuals without lifetime depression from the population-based BD cohort used as controls (data not shown). The analysis identified only three genes with CM interactions that overlapped the meta-analysis findings in GS. These were the killer cell immunoglobulin-like receptors *KIR3DL1* (FDR = 0.0018) and *KIR3DL2* (FDR = 0.0011), expressed in natural killer cells and subsets of T cells, and the stress-activated mitogen-activated protein kinase-activated protein kinase 2, *MAPKAPK2* (FDR = 0.031). The differences observed between BD analyses utilizing the population-based cohort only, or the depression cohort compared to controls, appeared to arise from small genetic differences (i.e. nominal associations in 110 of the studied genes, and nearly significant differences in the polygenic scores for ICD 10-defined depression, p = 0.059, as well as for self-harm behavior, p = 0.073) between individuals within the depression cohort (i.e. moderate to severe depression, with hospitalization) and those with lifetime depression in the population-based cohort (i.e. milder forms of depression, without hospitalization). This further suggests effects of immune-CM interactions on depression severity, which may go in line with previous reports of differential effects of trauma types on immunity and psychopathology [28, 29].

Taken together, the results from our depression and CM interaction analyses converged in genes and processes relevant for hematopoiesis. There is evidence that chronic stress shifts hematopoietic stem cell differentiation toward a primed myeloid cell lineage [42]. Therefore, we believe it is plausible that combinations of immune genetic profiles and types of early life stressors interact in specific manner to promote permanent hematopoietic imbalances that lead to chronic changes in immune and stress responses, and contribute to psychiatric illness later in life.

In conclusion, our study provides insights into the relationship between immunity, CM and depression, as well as a number of candidate genes that may aid in the investigations of potential therapeutic approaches to prevent and treat depression in general and, in particular, in individuals who experienced CM. We acknowledge that our study has limitations, including 1) a relatively small sample size, particularly for the CM interaction analysis, 2) a broad phenotype definition, including mostly individuals with self-reported lifetime depression, 3) a large proportion of related individuals (up to second degree family members) in the discovery sample, and 4) an incomplete representation of the majority of the immune pathways studied (i.e. 47.8 - 100% of pathway genes were present in the datasets). We aimed to overcome the sample size and kinship issues by performing the CM interaction analysis in an independent sample of non-related individuals and placing emphasis on the overlapping findings. Also, focusing on selected pathways helped reduce the burden of multiple testing in comparison to using our full ImmuneSet for these investigations. Power calculations suggested that our CM samples should be able to detect interactions with relatively large effects (power: 80%, alpha: 0.05, effect: 0.1; data not shown). Moreover, we aimed to compensate for the incomplete representation of pathways by conducting a PPI network analysis of the most robust (i.e. replicated) gene-level findings. Future studies should evaluate the identified associations using more refined phenotype definitions and larger samples.

## Supplementary information

Supplementary Tables

## Data Availability

All data supporting the findings of this study are available within the paper and its Supplementary Information. The datasets analyzed in this study are accessible through the GS website and KB, but restrictions apply.
